# Surveillance for foodborne disease outbreaks in Zhejiang Province, China, 2015–2020

**DOI:** 10.1186/s12889-022-12568-4

**Published:** 2022-01-19

**Authors:** Lili Chen, Liang Sun, Ronghua Zhang, Ningbo Liao, Xiaojuan Qi, Jiang Chen

**Affiliations:** 1 Department of Nutrition and Food Safety, Zhejiang Provincial Center for Disease Control and Prevention, 3399 Binsheng Road, Binjiang District, Hangzhou City, 310051 Zhejiang Province China; 2 College of Food Science and Engineering, Jiangxi Agricultural University, Nanchang, 330045 China

**Keywords:** Foodborne disease outbreak, *Vibrio parahaemolyticus*, Poisonous mushroom

## Abstract

**Background:**

Foodborne diseases are a major cause of morbidity and mortality and a major public health problem worldwide. We aim to explore characteristics of foodborne disease outbreaks (FBDOs) in Zhejiang Province and to provide data support for foodborne disease prevention and control. To our knowledge, few such analyses have been published at the provincial level.

**Methods:**

Descriptive statistical methods were used to analyze the data reported by centers for disease control (CDC) at all levels in Zhejiang Province through Foodborne Disease Outbreaks Surveillance System (FDOSS) during 2015–2020.

**Results:**

A total of 962 FBDOs were reported during this period, resulting in 8324 illnesses, 1028 hospitalizations, and 20 deaths. The number of outbreaks (410 outbreaks, 42.62%) and cases (4991 cases, 59.96%) caused by bacteria were the largest, followed by poisonous mushrooms (157 outbreaks, 587 cases), which was the main cause of death (15 deaths, 75%). The highest number of FBDOs occurred in households (381 outbreaks, 39.60%), followed by restaurants (219 outbreaks, 22.77%) and canteens (174 outbreaks, 18.08%). Animal-based foods were the most common single food reported (232 outbreaks, 24.12%), followed by poisonous mushrooms (162 outbreaks, 16.84%), and plant-based foods (133 outbreaks, 13.83%). Poisonous mushrooms took the first place in outbreaks in households (38.32%, 146/381), while bacteria took the first place in outbreaks outside households. *Vibrio parahaemolyticus* was responsible for the largest number of outbreaks (232 outbreaks, 24.12%), which mainly occurred in catering service units (93.10%, 216/232). Different types of bacteria tended to be found in different food categories, such as *Vibrio parahaemolyticus*, which was mainly found in aquatic products.

**Conclusions:**

Analysis of FBDOs can provide insight into the most important pathogens and sources of foodborne disease, helping authorities identify high-risk etiologies, high-risk foods, and high-risk settings to guide policies that would reduce FBDOs.

**Supplementary Information:**

The online version contains supplementary material available at 10.1186/s12889-022-12568-4.

## Background

Foodborne diseases are a major cause of morbidity and mortality and a major obstacle to socio-economic development worldwide. The World Health Organization estimates that 600 million people worldwide fall ill from eating contaminated food each year, resulting in 420,000 deaths and a loss of 33 million healthy life years [1]. In low-and middle-income countries, food insecurity costs $110 billion a year in lost productivity and lost health care costs [2]. As the largest developing country, the burden of foodborne diseases in China is not optimistic. A nationwide survey of acute gastroenteritis estimates (AGI) that 748 million AGI cases and 420 million medical consultations occur in China each year [3]. Therefore, we need early identification, monitoring and early warning through foodborne disease surveillance systems to identify trends, risk factors and disease burden of specific diseases in order to reduce foodborne diseases. Since 1996, the United States has successively established the laboratory-based Foodborne Disease Outbreak Surveillance Network (FDOSS), foodborne Disease Active Surveillance Network (FoodNet), National Foodborne Disease Molecular Typing Network (PulseNet) and other surveillance systems have been successfully applied in the identification, investigation, tracing, early warning of foodborne disease outbreaks (FBDOs) [4–6].

China has established a web-based foodborne disease surveillance platform since 2011, which has gradually played a role in FBDOs, early warning of sudden food safety incidents and research on foodborne disease burden. The platform mainly includes: the Foodborne Disease Outbreaks Surveillance System (FDOSS), the Foodborne Disease Surveillance and Reporting System (FDSRS), the National Molecular Traceability Network for Foodborne Diseases (TraNet) and other surveillance systems. The China National Center for Food Safety Risk Assessment (CFSA) maintains and manages the platform for data collection and periodic reporting to the National Health Commission [4, 5]. Through the collection and analysis of FBDOs from 3378 CDCs (as of June 2017, the mid-point of 2015–2020) by FDOSS, we can master the high-risk foods and risk factors of FBDOs, and provide a scientific basis for the government to formulate and adjust the prevention and control strategies of foodborne disease. Through surveillance of case information and specific pathogens by FDSRS, we can discover clusters in time, improve the ability of early identification, early warning and prevention and control of food safety risks, and master the incidence baseline of important foodborne diseases. TraNet is based on molecular typing and cluster analysis of foodborne pathogenic bacteria isolates from patients and food to identify clustered cases and guide the traceability investigation of pathogenic foods. After years of efforts, the rate of timely handling and reporting of FBDOs has increased significantly, and the rate of concealment and omission has decreased [4].

Located in the southeast coast of China, Zhejiang province has a permanent population of 58.5 million at the end of 2019, with a GDP of 6235.74 billion yuan and per capita GDP of 107,624 yuan, ranking the fourth in China [7]. In order to summarize epidemiological characteristics of FBDOs and provide effective interventions to prevent FBDOs in Zhejiang province, we analyzed the surveillance data of FBDOs in Zhejiang province from 2015 to 2020.

## Methods

### Outbreak definition

A foodborne disease outbreak (FBDO) is defined as two or more cases of a similar illness resulting from ingestion of a common food [8]. Diagnostic criteria and principles of management for FBDO of different etiologies were issued by the ministry of health in 1996 and have been used in outbreak investigation ever since [9]. Outbreaks that did not meet these criteria were not reported to the FDOSS.

### Data source

From 2015 to 2020 inclusive, outbreak reports were reported passively to FDOSS from 11 prefecture-level CDCs and more than 80 county-level CDCs in Zhejiang Province. And they investigate FBDOs and report data to FDOSS using a standard form. The information collected for each outbreak includes reporting region, date of occurrence, setting, etiology, food categories, number of illnesses / hospitalizations / deaths, and some other details. Unknown etiology refer to those FBDOs where the confirmed etiology has not been identified. If more than one etiologic agent is reported in a FBDO, the etiology of the outbreak is categorized as multiple etiologies. Settings of food prepared or consumed were classified into 11 categories, including household, restaurant, staff canteen, school canteen, rural banquet, chophouse, retail food outlets, school, deliver meals, fast food restaurant, street stall. The setting that cannot be determined was classified as “Unknown location”. Catering service units refers to all commercial food settings, such as restaurants, staff canteens, rural banquets, chophouse and fast food restaurants, etc.

### Statistical analysis

Data was exported from the FDOSS and analyzed in Excel 2013. Population data of prefectures and counties in Zhejiang Province are from 2015 to 2020 statistical year book of Zhejiang Province. The GIS map data of Zhejiang Province is downloaded by the national basic geographic information center of China (http://ngcc.sbsm.gov.cn). ArcGis10.2 software [10] was used to making thematic map. We used the average of the total population of Zhejiang Province from 2015 to 2019 as the denominator to calculate the per capita rate of FBDOs .

## Results

### General characteristics

**Fig. 1:**
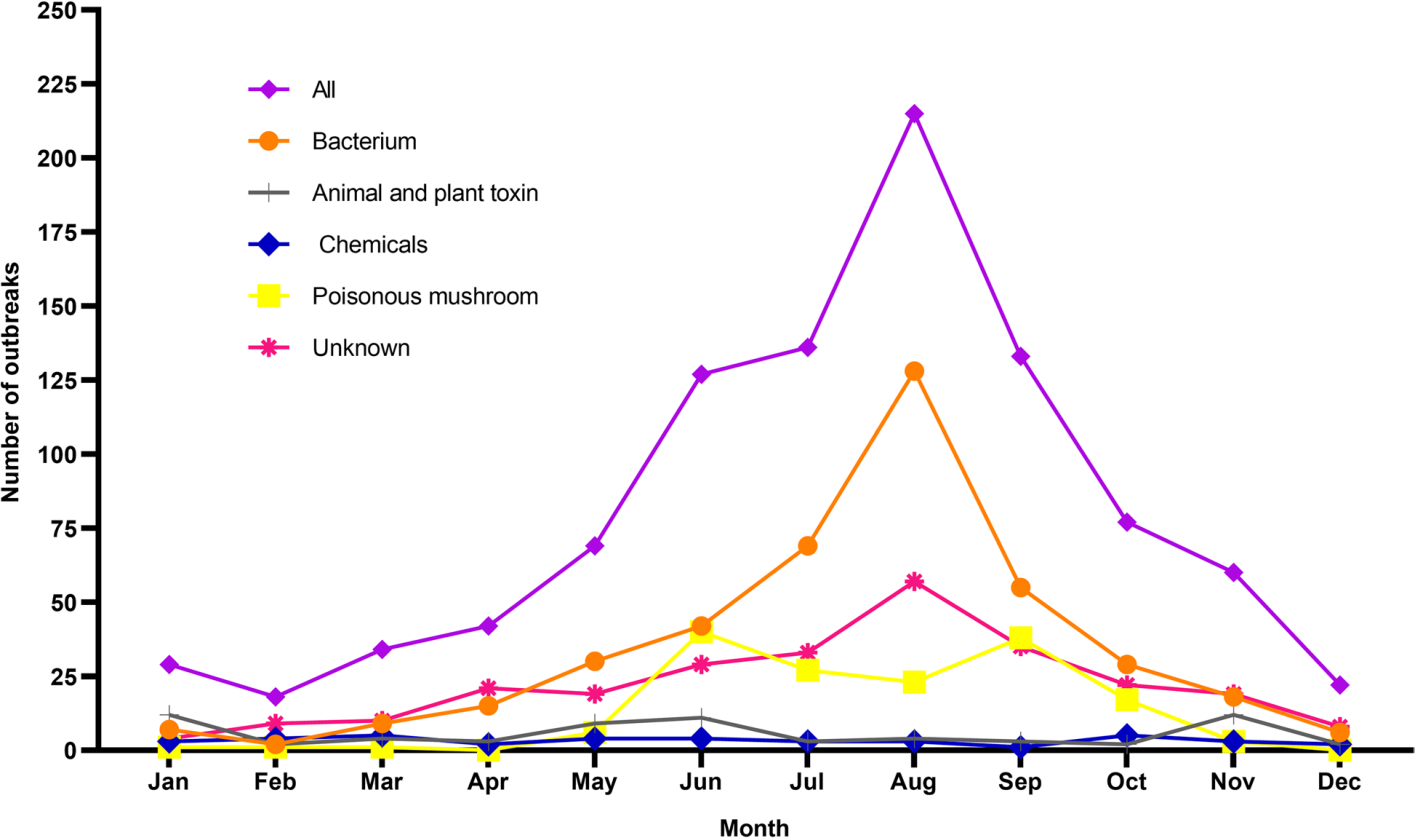
Seasonality of foodborne disease outbreaks in Zhejiang, by etiology, 2015–2020

Between 2015 and 2020, a total of 962 FBDOs were reported, resulting in 8324 illnesses, 1028 hospitalizations, and 20 deaths (Table 1). The number of reported outbreaks was the highest in 2020 and the lowest in 2015, with an average of 161 outbreaks per year. The average number of outbreaks and outbreak-associated cases per 1 million population in all 11 prefectures for the six-year period were 3.25 and 167.95. FBDOs were reported in 11 prefectures in Zhejiang Province, ranging from 45 outbreaks (Huzhou) to 173 outbreaks (Hangzhou) (Fig.S1). The seasonal characteristics of FBDOs were obvious, with high incidence in summer and autumn, and the peak of outbreaks was in August (215 cases, 22.35%). The seasonal trends of microbial outbreaks and outbreaks of unknown etiology were similar to that of the overall FBDOs; there was a seasonal variation trend of mushroom poisoning, with the peak period from June to October; the seasonal trends of outbreaks of other etiologies were not obvious (Fig. 1).

**Table 1:** Number of reported foodborne disease outbreaks, cases, hospitalizations, and deaths, by year, Zhejiang, 2015–2020

Year	Outbreaks		Illnesses		Hospitalizations		Deaths	
	Number	%	Number	%	Number	%	Number	%
2015	115	11.95	988	11.87	115	11.19	10	50.00
2016	163	16.94	1463	17.58	257	25.00	3	15.00
2017	124	12.89	983	11.81	91	8.85	0	0.00
2018	172	17.88	1427	17.14	192	18.68	1	5.00
2019	187	19.44	1698	20.40	235	22.86	5	25.00
2020	201	20.89	1765	21.20	138	13.42	1	5.00
Total	962	100.00	8324	100.00	1028	100.00	20	100.00

### Etiology

The classification of pathogenic factors of FBDOs in Zhejiang Province mainly includes bacteria, poisonous mushrooms, plant toxins, animal toxins, chemical agents, virus, and multiple etiologies. The number of FBDOs and cases caused by various etiologies varied greatly (Table 2). Etiology was identified for 72.35% of the reported outbreaks, and the single etiology accounted for 72.04% of all outbreaks. Among the identified causes of FBDOs (696 outbreaks, 6528 cases), the number of outbreaks (410 outbreaks, 42.62%) and cases (4991 cases, 59.96%) caused by bacteria were the largest, followed by poisonous mushrooms (157 outbreaks, 587 cases). A total of 20 deaths were reported, which were caused by poisonous mushrooms (15 deaths), bacteria (3 deaths) and poisonous plants (2 deaths).

**Table 2:** Number and percentage of foodborne disease outbreaks, illnesses, hospitalizations, and deaths, by etiology, Zhejiang Province, 2015–2020

Etiology	Outbreaks		Illnesses		Hospitalizations		Deaths	
	Number	%	Number	%	Number	%	Number	%
**Bacterial**	**410**	**42.62**	**4991**	**53.88**	**546**	**46.1**	**3**	**15**
*Vibrio parahaemolyticus*	232	24.12	2472	26.69	181	15.29	1	5
*Salmonella*	81	8.42	1032	11.14	231	19.51	0	0
*Diarrheogenic Escherichia coli*	25	2.6	727	7.85	6	0.51	0	0
*Staphylococcus aureus*	24	2.49	288	3.11	67	5.66	0	0
*Bacillus cereus*	21	2.18	200	2.16	34	2.87	1	5
others	15	1.56	183	1.98	8	0.68	0	0
*Proteusbacillus vulgaris*	8	0.83	57	0.62	17	1.44	0	0
*Pseudomonas cocovenenans subsp farinofermantans*	2	0.21	4	0.04	2	0.17	1	5
*Aeromonas*	2	0.21	28	0.3	0	0	0	0
**Poisonous mushroom**	**162**	**16.84**	**607**	**6.55**	**210**	**17.74**	**15**	**75**
**Plant toxins**	**53**	**5.51**	**365**	**3.94**	**52**	**4.39**	**2**	**10**
Undercooked Phaseolus/Saponin	14	1.46	177	1.91	20	1.69	0	0
Tung oil or seed	5	0.52	31	0.33	2	0.17	0	0
Aconitine	3	0.31	19	0.21	10	0.84	0	0
Bitter bottle gourd	8	0.83	46	0.5	1	0.08	0	0
Colchicine	4	0.42	23	0.25	0	0	0	0
Datura/hyoscyamine	2	0.21	6	0.06	5	0.42	0	0
Others	17	1.77	67	0.72	14	1.18	2	10
**Animal toxins**	**17**	**1.77**	**72**	**0.78**	**12**	**1.01**	**0**	**0**
Tetrodotoxin	7	0.73	17	0.18	9	0.76	0	0
Histamine	3	0.31	35	0.38	1	0.08	0	0
Others	7	0.73	20	0.22	2	0.17	0	0
**Chemical agents**	**37**	**3.85**	**223**	**2.41**	**89**	**7.52**	**0**	**0**
Nitrite	20	2.08	157	1.69	67	5.66	0	0
Pesticides	5	0.52	27	0.29	5	0.42	0	0
Lead	8	0.83	30	0.32	10	0.84	0	0
Veterinary drugs	3	0.31	7	0.08	6	0.51	0	0
Methanol	1	0.1	2	0.02	1	0.08	0	0
**Virus**	**20**	**2.08**	**279**	**3.01**	**3**	**0.25**	**0**	**0**
Norovirus	15	1.56	195	2.11	3	0.25	0	0
Others	5	0.52	84	0.91	0	0	0	0
**Multiple etiologies**	**3**	**0.31**	**11**	**0.12**	**0**	**0**	**0**	**0**
**Unknown etiology**	**260**	**27.03**	**1772**	**19.13**	**116**	**9.8**	**0**	**0**
**Total**	**962**	**100.00**	**9263**	**100.00**	**1184**	**100.00**	**20**	**100**

*Vibrio parahaemolyticus* (56.59%, 232/410) was the most common pathogenic bacteria in Zhejiang Province, followed by *Salmonella* (19.76%, 81/410). Serotyping has been performed in 121 outbreaks caused by *Vibrio parahaemolyticus*, with O3K6 serotype accounting for the largest proportion (81.82%, 99/121), followed by O4K8 serotype (9.09%, 11/121). A total of 70 outbreaks caused by *Salmonella* have been serotyped. The most common serotype was *Salmonella Enteritidis* (52.86%, 37/70), followed by *Salmonella Typhimurium* (17.14%, 12/70) and *Salmonella* Blegdam (4.29%,3/70). Three of the 20 deaths were caused by pathogenic bacteria, including *Vibrio parahaemolyticus* (1 death), *Bacillus cereus* (1 death) and *Pseudomonas cocovenenans subsp farinofermantans* (producing bongkrekic acid, 1 death). Poisonous mushroom was the most common cause of deaths, accounting for 75% of deaths. Nine deaths from four outbreaks were presumed to have been caused by *Amanita Rimosa* or *A. fuliginea*. Another death in an outbreak was caused by *Russula Subnigricans.* Poisonous mushrooms were mainly collected from the wild by patients or relatives and friends.

Undercooked phaseolus (26.92%, 14/52), bitter bottle gourd (15.38%, 8/52) and tung oil or seed (9.62%, 5/52) are the most common plant toxins. The identified animal toxins mainly include tetrodotoxin (41.18%, 7/17) and histamine (17.65%, 3/17). Nitrite (54.05%, 20/37) was the most common chemical agent responsible for FBDOs, followed by lead (21.62%, 8/37) and pesticides (13.51%, 5/37). The proportion of viruses (2.08%) causing FBDOs is relatively small, mainly Norovirus (75%, 15/20).

### Setting

Confirmed settings were provided in the majority (99.17%) of the outbreak reports (Table 3). The highest number of outbreaks occurred in households (381 outbreaks, 39.60%), followed by restaurants (219 outbreaks, 22.77%) and canteens (174 outbreaks, 18.08%). Households were responsible for the majority of deaths (18 deaths, 90%) while FBDOs in restaurants resulted in the highest number of cases (2185 cases, 26.25%). Of the 18 deaths in households, 15 were caused by poisonous mushrooms, and the remaining three deaths were caused by Pokeberry root (1 death), *Pseudomonas cocovenenans* (1 death), and *Bacillus cereus* (1 death), respectively.

**Table 3:** Number of reported foodborne disease outbreaks, cases, and deaths, by setting, Zhejiang Province, 2015–2020

Etiology	Outbreaks		Illnesses		Hospitalizations		Deaths	
	Number	%	Number	%	Number	%	Number	%
Household	381	39.60	1365	16.40	362	35.21	18	90.00
Restaurant	219	22.77	2185	26.25	224	21.79	2	10.00
Staff canteen	112	11.64	1433	17.22	135	13.13	0	0.00
School canteen	63	6.55	1404	16.87	56	5.45	0	0.00
Rural banquet	50	5.20	679	8.16	63	6.13	0	0.00
Chophouse	34	3.53	183	2.20	14	1.36	0	0.00
Retail food outlets	31	3.22	312	3.75	63	6.13	0	0.00
School	23	2.39	401	4.82	95	9.24	0	0.00
Deliver meals	16	1.66	102	1.23	12	1.17	0	0.00
Fast food restaurant	13	1.35	131	1.57	1	0.10	0	0.00
Street stall	12	1.25	62	0.74	1	0.10	0	0.00
Unknown location	8	0.83	67	0.80	2	0.19	0	0.00
Total	962	100.00	8324	100.00	1028	100.00	20	100.00

### Food

Confirmed foods were reported for 662 (68.81%, 662/962) of the 962 outbreaks and 557 (57.90%, 557/962) outbreaks were attributed to a single food (Table S1). Animal-based foods were the most common single food reported (41.65%, 232/557), followed by poisonous mushrooms (29.08%, 162/557), and plant-based foods (23.88%, 133/557). In the category of animal-based foods, aquatic products was the most common food reported (59.48%, 138/232), followed by meat and meat products (34.48%, 80/232). In the category of plant-based foods, vegetables were the most common food reported (27.07%, 36/133), followed cereals (23.31%, 31/133) and flour products (19.55%, 26/133).

### Setting and etiology

Outbreaks caused by different etiologies have different setting distributions (Fig.S2). The outbreaks caused by bacteria occurred in all settings, and accounted for the largest proportion in each setting other than households. The outbreaks caused by poisonous mushrooms (90.12%,146/162), plant toxins (54.72%, 29/53), animal toxins (88.24%, 15/17) and chemical agents (81.08%, 30/37) mainly occurred in households.

Further analysis found that FBDOs caused by *Vibrio parahaemolyticus* mainly occurred in catering service units (93.10%, 216/232), such as restaurants, staff canteens, rural Banquets, chophouse and fast food restaurants, etc. The proportion of outbreaks caused by *Salmonella* in households and catering service units was 45.68% (37/81) and 54.32% (44/81), respectively.

### Food and etiology

Different foods were involved in outbreaks caused by different etiologies (Fig. S3). The most common single food that caused bacterial FBDOs included aquatic products (21.22%, 87/410) and meat and meat products (11.71%, 48/410) in animal-based foods, as well as flour products (4.39%, 18/410) and cereals (4.15%,17/410) in plant-based foods. Further analysis showed that the main pathogenic factor of aquatic products was *Vibrio parahaemolyticus* (54.35%, 75/138). *Salmonella* (18.99%, 15/79) and *Vibrio parahaemolyticus* (18.99%, 15/79) were the main pathogenic factors in meat and meat products. *Bacillus cereus* (32.26%, 10/31) accounted for the largest proportion of outbreaks caused by cereals, while *Salmonella* also accounted for the most in flour products (46.15%, 12/26) and eggs (50.00%, 4/8). Animal toxins were only found in aquatic products (100%, 17/17), such as puffer fish, snail, grouper, etc. Plant toxins were found in phaseolus (26.42%,14/53), bottle gourd (15.09%, 8/53) and other plants. The foods involved in outbreaks caused by chemical agents mainly included plant-based foods and other foods (liquor and condiments). Nitrites (56.25%, 9/16) and pesticides (31.25%, 5/16) were the main pathogenic factors of plant-based foods. Outbreaks caused by liquor were mainly caused by the presence of lead in the containers (85.71%, 6/7). The outbreaks caused by condiments were mainly caused by the misuse of nitrites (75%, 6/8).

## Discussion

The average number of outbreaks and cases between 2015 and 2020 are significantly increased, higher than 1.1 outbreaks per 1 million and 28.6 cases per 1 million reported by Yong-ning Wu et al. [11] from 2003 to 2008. The increase in the number of reports is related to the upgrading of the operating environment of the surveillance system and the optimization of the reporting process as well as the improvement of regional awareness of reporting.

According to our study, all the bacteria together are responsible for the largest number of FBDOs in Zhejiang Province, which is consistent with the studies in Mainland China [12], the United States [13], the Republic of Korea [14] and Brazil [15]. The number of outbreaks caused by *Vibrio parahaemolyticus* ranks first among all etiologies, which is consistent with the studies in China’s coastal provinces such as Hainan [16] and Qingdao [17], but quite different from those in some inland provinces, such as Henan [18] and Yunnan [19]. A review of 2447 literatures in China also found that in littoral domain, *Vibrio parahaemolyticus* caused the most outbreaks, whereas in inland domain, the largest percentage of outbreaks was caused by *Salmonella* [20]. Therefore, there are regional differences in the distribution of pathogenic bacteria in China. A study in the United States [21] also showed regional differences in FBDOs caused by *Salmonella Enteritidis* between 1990 and 2015. These studies suggested that region-specific policies should be introduced to reduce FBDOs.

In outbreaks caused by *Vibrio parahaemolyticus*, the largest number of food categories involved were aquatic products (32.32%, 75/232), including mollusks, crustaceans, and fish. Zhejiang Province is a coastal province with a vast sea area and abundant aquatic products. A study showed that the detection rate of *Vibrio parahaemolyticus* in raw/semi-raw animal aquatic products in Zhejiang Province was as high as 32.52% [22]. Therefore, consumers are advised to avoid eating raw or undercooked aquatic products as much as possible to reduce the risk of disease caused by *Vibrio parahaemolyticus*. However, avoiding all raw seafood can be difficult for those who are in the habit of eating raw or semi-raw shellfish and other seafood. A study from the United States showed that the oysters associated with the outbreak were harvested when the average daily water temperature exceeded 15.0 °C, suggesting that the harvest water temperature may play a role in *Vibrio parahaemolyticus* growth [23]. Therefore, local residents with raw food habits are suggested cooking shellfish in warm months instead of a total avoidance of raw shellfish. In addition, outbreaks caused by *Vibrio paraolyticus* have involved meat and meat products, multiple foods and mixed foods, mainly due to cross-contamination. Another factor that affected the occurrence of *Vibrio parahaemolyticus* outbreaks is the location of food consumption, which was similar to a study on *Campylobacter* outbreaks [24]. Given that outbreaks caused by *Vibrio parahaemolyticus* mainly occurred in catering service units (93.10%, 216/232), we believe that enhanced regulation and specific training for managers and chefs in catering service units could significantly reduce outbreaks caused by *Vibrio parahaemolyticus*. The training can include the following aspects: the seafood should be cooked thoroughly; containers and hands handling raw seafood should be thoroughly washed before handling other food, especially ready-to-eat food; ready-to-eat cooked meat should be prevented from cross-contamination and served within a specified time after it has been cut, otherwise it should be quickly cooled and refrigerated; restaurants without processing and supply capacity are not allowed to serve cold dishes and raw ready-to-eat seafood, etc.

An American study has shown that retail food service establishments accounted for more *Salmonella enterica* outbreaks than any other food preparation setting during 1973–2009 [25]. However, our study found a high percentage of *Salmonella* in households (45.68%, 37/81) and food service units (54.32%, 44/81) in Zhejiang Province. This means that in addition to focusing on food service units, we should also raise awareness of household prevention of foodborne diseases. *Salmonella*-related outbreaks have involved foods such as meat and meat products, flour products and eggs. Meat and meat products included chicken, duck, pork and beef. These meats were cold cooked meats that were not reheated before being eaten. Flour products included cold-processed cakes and sandwiches (often containing eggs and meat). Outbreaks were most frequently attributed to cross-contamination and improper storage temperature. When we come up with prevention and control strategies, we should not ignore other foods besides chicken and eggs.

Poisonous mushrooms are the second major cause of FBDOs and the main cause of FBDO-associated death in Zhejiang Province. The number of outbreaks caused by poisonous mushrooms increased from 13 per year during 2015–2017 to 41 per year during 2018–2020. The increasing number of outbreaks was largely linked to increased awareness of reporting after extensive publicity about mushroom poisoning and training of doctors. The analysis found that outbreaks caused by poisonous mushrooms mostly occurred in households (90.12%, 146/162), and mainly in rural households. Patients or their relatives and friends picked mushrooms and ate them at home after cooking, resulting in poisoning. A small amount of mushrooms were also purchased from small vegetable markets or roadside stalls. In view of the poisoning caused by farmers’ picking and eating by themselves, it is necessary to strengthen the publicity and education for key groups. As there are many kinds of poisonous mushrooms, and some poisonous mushrooms are very similar to non-toxic mushrooms, it is sometimes difficult to distinguish them only by their morphology. The identification of poisonous mushroom is mainly based on expert identification of external morphology, microscopic characteristics and DNA molecular markers [26–28]. It is difficult for the general public to tell if mushrooms are poisonous, so it is advised not to pick, buy or eat wild mushrooms. For small vegetable markets or roadside stalls, authorities should strengthen supervision and ban the sale of wild mushrooms from unknown sources.

Finally, we would like to briefly discuss the impact of COVID-19 on the surveillance of FBDOs in 2020. The number of FBDOs reported in 2020 was the highest in the last 6 years. This may be due to the fact that catering service units in Zhejiang reopened at the end of February 2020 after being closed during the first wave of the pandemic, and no new outbreaks have occurred since then. Although the number of FBDOs has not decreased as a result of COVID-19 in general, the time distribution, pathogenic factors and settings of reported FBDOs were different from those of previous years. In previous years, the outbreak peak occurred in August. In 2020, the number of reported outbreaks showed an increasing trend from January to June. However, the number decreased rapidly in July and reached the second peak in August. The rapid decline in the number of outbreaks in July may be related to a decrease in eating out due to the novel COVID-19 pandemic in Beijing (June) and extensive publicity by national authorities on how consumers can be good at food safety under the new epidemic. COVID-19 has also affected the distribution of outbreak settings. The number of FBDOs in restaurants decreased significantly. In contrast, the number of outbreaks in households, staff canteens and school canteens increased. According to previous monitoring data, the outbreaks caused b*y Vibrio parahaemolyticus* in Zhejiang mainly occurred in restaurants and other catering units. The proportion of outbreaks caused by *Vibrio parahaemolyticus* was significantly lower than in previous years due to a decrease in eating out. Overall, the COVID-19 pandemic in Beijing (June) did have some impact on FBDOs in Zhejiang Province.

Some limitations of this study need to be explained. The outbreak reporting rate has obviously improved since we collected FBDOs through FDOSS, but for some reason, such as the inability to conduct an epidemiological investigation due to lack of patient cooperation, there were still some under-reporting. In addition, the database is dynamic, and lower CDCs can modify and delete previous reports, as well as add reports, so the results of this analysis represent the data available at a single point in time, and may differ from data published before or after.

## Conclusions

Bacteria and poisonous mushrooms were the main causes of FBDOs in Zhejiang Province. *Vibrio parahaemolyticus* and *Salmonella* accounted for the largest proportion of bacteria. Due to the different epidemiological characteristics of FBDOs caused by different etiologies, We recommend taking targeted measures according to the characteristics of different etiologies, settings and food vehicles to improve the efficiency of prevention and control. Poisonous mushrooms were the leading cause of death from FBDOs in Zhejiang Province. Since the general public does not have a reliable way to identify poisonous mushrooms, we recommend not picking, buying or eating wild mushrooms. Most foodborne diseases are preventable, so the information provided by timely investigation, management and reporting of FBDOs has the potential to help reduce them. In the next step, we will further strengthen the surveillance of FBDOs, improve the identification rate of the causes of the epidemic, carry out attribution analysis, and provide data support for “precise prevention and control”.

## Supplementary Information

**Additional file 1: Fig. S1.** Number of reported foodborne disease outbreaks in Zhejiang, by prefecture, 2015–2020. **Fig. S2.** Proportion of the etiology of foodborne disease outbreaks in different settings, in Zhejiang, 2015–2020. **Fig. S3.** Proportion of the etiology of foodborne disease outbreaks in different food categories, in Zhejiang, 2015–2020.

**Additional file 2: Table S1.** Number of reported foodborne disease outbreaks, cases, and deaths, by food, Zhejiang Province, 2015–2020.

## Data Availability

The data that support the findings of this study are available from the Foodborne Disease Outbreaks Surveillance System of the China National Center for Food Safety Risk Assessment, and these data are not publicly available. The data that support the findings of this study are available from the Foodborne Disease Outbreaks Surveillance System (https://sppt.cfsa.net.cn/goto), and these data are not publicly available.

## Abbreviations

FBDOs: Foodborne disease outbreaks
CDC: Centers for disease control
AGI: Acute gastroenteritis estimates
FDOSS: Foodborne Disease Outbreaks Surveillance System
