# Supplementary material for: Surveillance for foodborne disease outbreaks in Zhejiang Province, China, 2015–2020
Source: BMC Public Health. 2022 Jan 19;22:135. doi: 10.1186/s12889-022-12568-4 (PMC8769373; doi:10.1186/s12889-022-12568-4)
Supplement: Supplementary file 1 — Additional file 1: Fig. S1. Number of reported foodborne disease outbreaks in Zhejiang, by prefecture, 2015–2020. Fig. S2. Proportion of the etiology of foodborne disease outbreaks in different settings, in Zhejiang, 2015–2020. Fig. S3. Proportion of the etiology of foodborne disease outbreaks in different food categories, in Zhejiang, 2015–2020. [file 12889_2022_12568_MOESM1_ESM.docx]

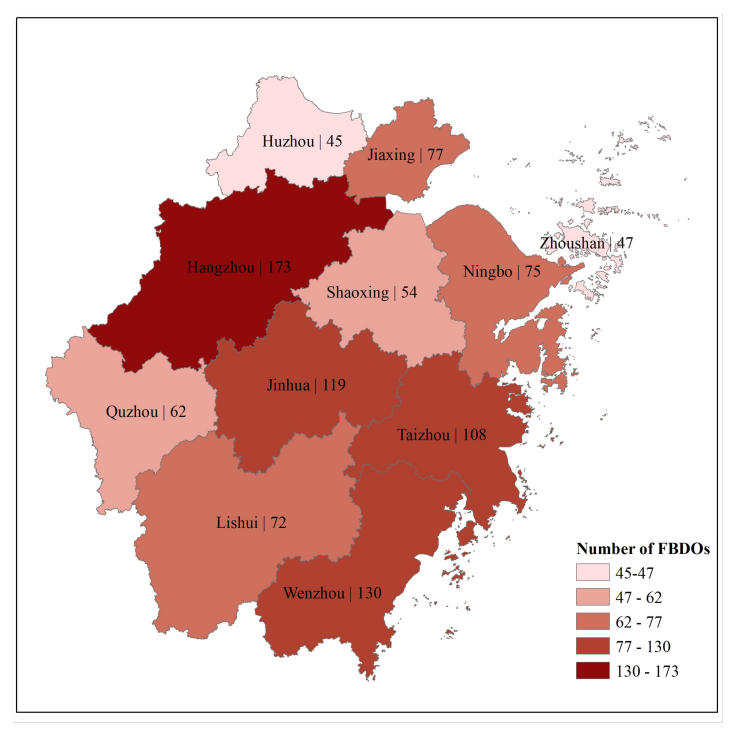


Fig. S1. Number of reported foodborne disease outbreaks in Zhejiang , by prefecture，2015–2020.


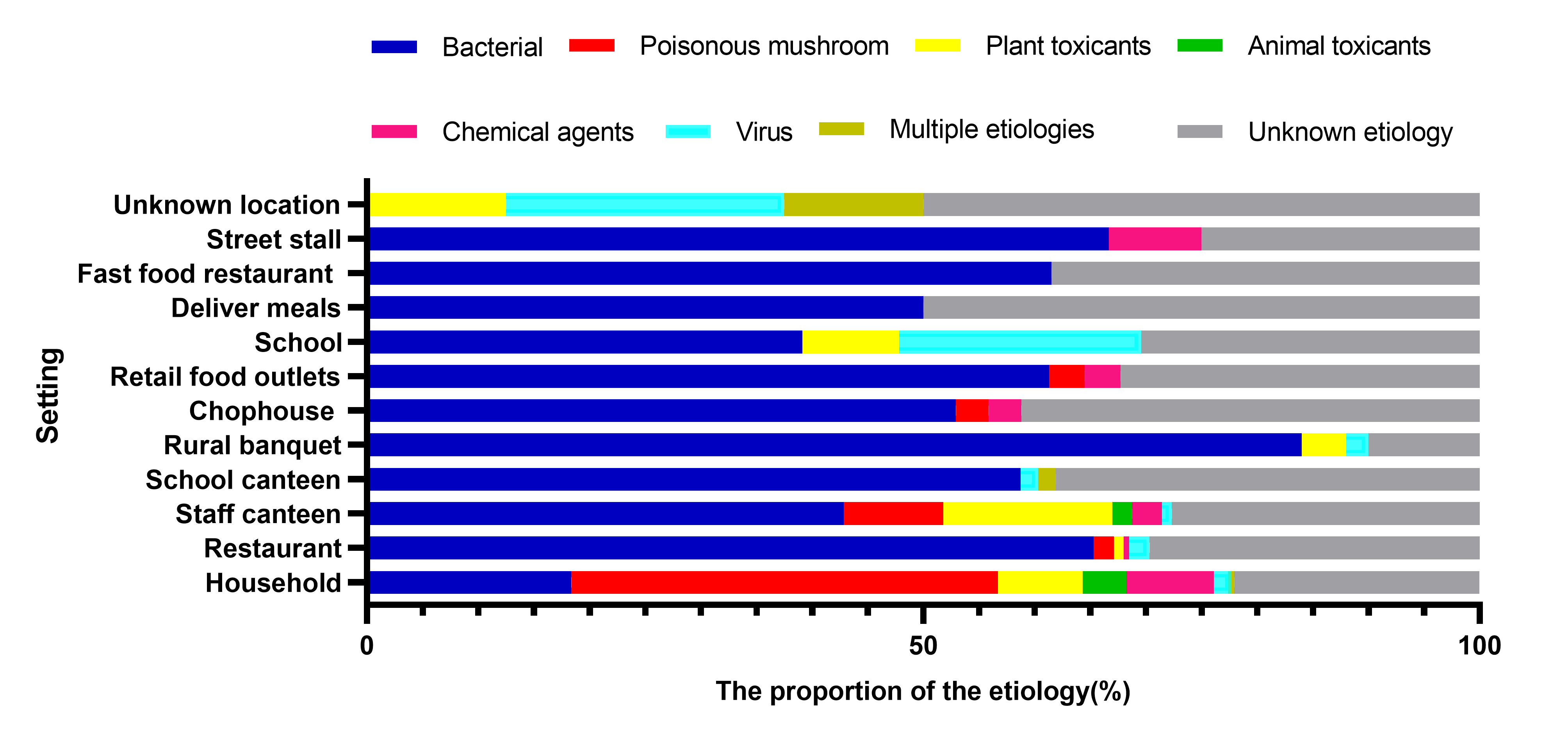


Fig. S2. Proportion of the etiology of foodborne disease outbreaks in different settings, in Zhejiang, 2015–2020


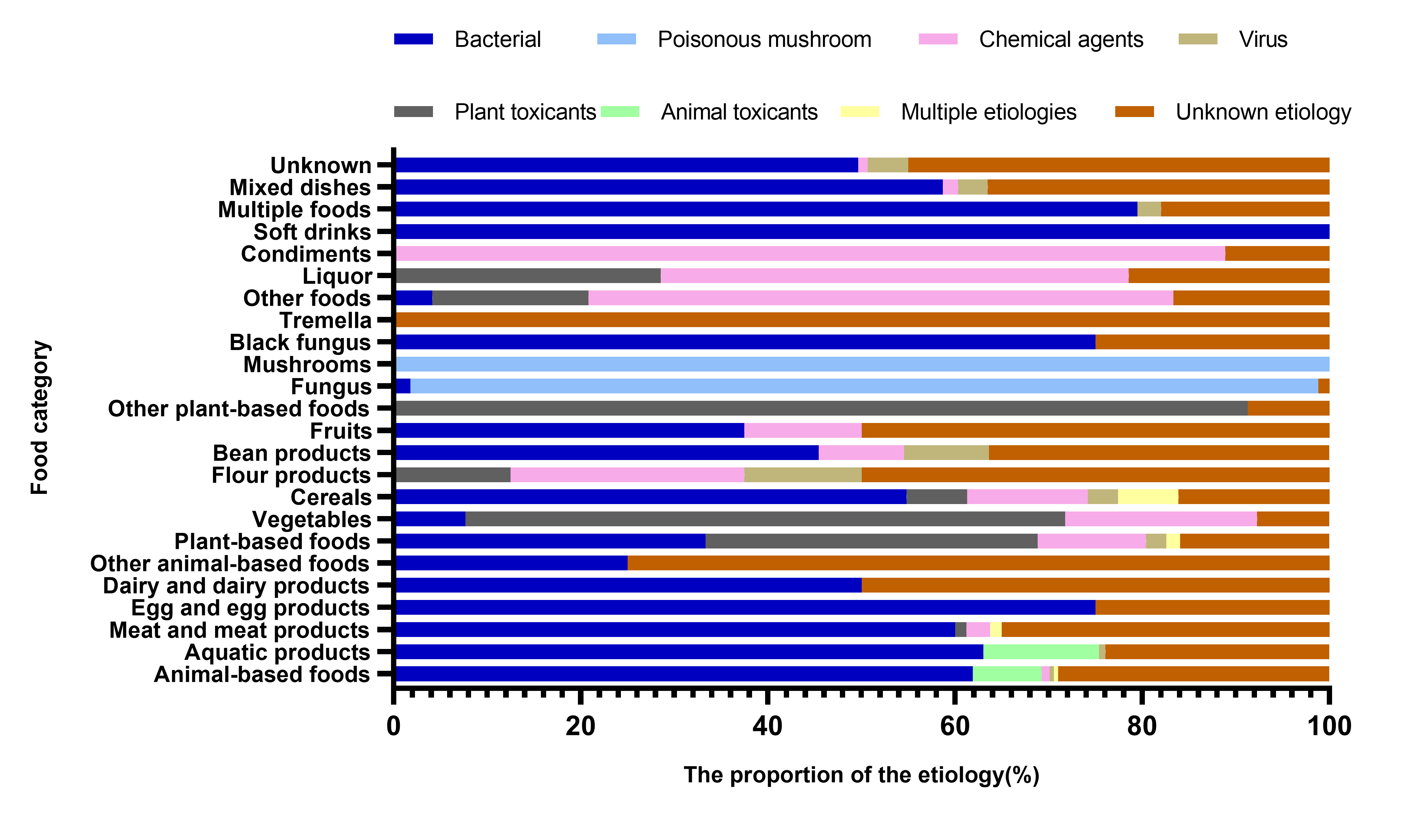


Fig. S3. Proportion of the etiology of foodborne disease outbreaks in different food categories, in Zhejiang, 2015–2020
