# Supplementary material for: Surveillance for foodborne disease outbreaks in Zhejiang Province, China, 2015–2020
Source: BMC Public Health. 2022 Jan 19;22:135. doi: 10.1186/s12889-022-12568-4 (PMC8769373; doi:10.1186/s12889-022-12568-4)
Supplement: Supplementary file 2 — Additional file 2: Table S1. Number of reported foodborne disease outbreaks, cases, and deaths, by food, Zhejiang Province, 2015–2020. [file 12889_2022_12568_MOESM2_ESM.docx]

Table S1 Number of reported foodborne disease outbreaks, cases, and deaths, by food , Zhejiang Province, 2015-2020.

| Food | | outbreaks | | | | illnesses | | | | hospitalizations | | | deaths | |
| --- | --- | --- | --- | --- | --- | --- | --- | --- | --- | --- | --- | --- | --- | --- |
|  |  | Number | | % | | Number | | % | | Number | | % | Number | % |
| **Animal-based foods** | **231** | | **24.01** | | **1950** | | **23.43** | | **152** | | **14.79** | | **0** | **0.00** |
| Aquatic products | 138 | | 14.35 | | 999 | | 12.00 | | 69 | | 6.71 | | 0 | 0.00 |
| Meat and meat products | 80 | | 8.32 | | 848 | | 10.19 | | 64 | | 6.23 | | 0 | 0.00 |
| Egg and egg products | 8 | | 0.83 | | 73 | | 0.88 | | 16 | | 1.56 | | 0 | 0.00 |
| Dairy and dairy products | 2 | | 0.21 | | 14 | | 0.17 | | 0 | | 0.00 | | 0 | 0.00 |
| Other animal-based foods | 4 | | 0.42 | | 20 | | 0.24 | | 3 | | 0.29 | | 0 | 0.00 |
| **Plant-based foods** | **138** | | **14.35** | | **1292** | | **15.52** | | **287** | | **27.92** | | **3** | **15.00** |
| Vegetable | 39 | | 4.05 | | 333 | | 4.00 | | 48 | | 4.67 | | 1 | 5.00 |
| Cereals | 31 | | 3.22 | | 308 | | 3.70 | | 83 | | 8.07 | | 2 | 10.00 |
| Flour products | 26 | | 2.70 | | 391 | | 4.70 | | 145 | | 14.11 | | 0 | 0.00 |
| Bean products | 11 | | 1.14 | | 134 | | 1.61 | | 0 | | 0.00 | | 0 | 0.00 |
| Fruits | 8 | | 0.83 | | 21 | | 0.25 | | 4 | | 0.39 | | 0 | 0.00 |
| Other plant-based foods | 23 | | 2.39 | | 105 | | 1.26 | | 7 | | 0.68 | | 0 | 0.00 |
| **Fungus** | **167** | | **17.36** | | **632** | | **7.59** | | **213** | | **20.72** | | **16** | **80.00** |
| [mushroom](../../../../Program Files (x86)/Youdao/Dict/8.9.6.0/resultui/html/index.html" \l "/javascript:;" \o ") | 162 | | 16.84 | | 607 | | 7.29 | | 210 | | 20.43 | | 15 | 75.00 |
| black fungus | 4 | | 0.42 | | 10 | | 0.12 | | 3 | | 0.29 | | 1 | 5.00 |
| [tremella](../../../../Program Files (x86)/Youdao/Dict/8.9.6.0/resultui/html/index.html" \l "/javascript:;" \o ") | 1 | | 0.10 | | 15 | | 0.18 | | 0 | | 0.00 | | 0 | 0.00 |
| **Other foods** | **24** | | **2.49** | | **110** | | **1.32** | | **54** | | **5.25** | | **0** | **0.00** |
| Liquor | 14 | | 1.46 | | 59 | | 0.71 | | 23 | | 2.24 | | 0 | 0.00 |
| condiments | 9 | | 0.94 | | 38 | | 0.46 | | 31 | | 3.02 | | 0 | 0.00 |
| Soft drinks | 1 | | 0.10 | | 13 | | 0.16 | | 0 | | 0.00 | | 0 | 0.00 |
| **Multiple foods** | **39** | | **4.05** | | **582** | | **6.99** | | **28** | | **2.72** | | **0** | **0.00** |
| **Mixed dishes** | **63** | | **6.55** | | **654** | | **7.86** | | **104** | | **10.12** | | **0** | **0.00** |
| **Unknown** | **300** | | **31.19** | | **3104** | | **37.29** | | **190** | | **18.48** | | **1** | **5.00** |
| **Total** | **962** | | **100.00** | | **8324** | | **100.00** | | **1028** | | **100.00** | | **20** | **100.00** |
